# High-throughput screening of SARS-CoV-2 main and papain-like protease inhibitors

**DOI:** 10.1093/procel/pwac016

**Published:** 2022-09-28

**Authors:** Yi Zang, Mingbo Su, Qingxing Wang, Xi Cheng, Wenru Zhang, Yao Zhao, Tong Chen, Yingyan Jiang, Qiang Shen, Juan Du, Qiuxiang Tan, Peipei Wang, Lixin Gao, Zhenming Jin, Mengmeng Zhang, Cong Li, Ya Zhu, Bo Feng, Bixi Tang, Han Xie, Ming-Wei Wang, Mingyue Zheng, Xiaoyan Pan, Haitao Yang, Yechun Xu, Beili Wu, Leike Zhang, Zihe Rao, Xiuna Yang, Hualiang Jiang, Gengfu Xiao, Qiang Zhao, Jia Li

**Affiliations:** The National Center for Drug Screening, Shanghai Institute of Materia Medica, Chinese Academy of Sciences, Shanghai 201203, China; State Key Laboratory of Drug Research and CAS Key Laboratory of Receptor Research, Shanghai Institute of Materia Medica, Chinese Academy of Sciences, Shanghai 201203, China; School of Pharmaceutical Science and Technology, Hangzhou Institute for Advanced Study, UCAS, Hangzhou 310024, China; School of Chinese Materia Medica, Nanjing University of Chinese Medicine, Nanjing 210023, China; University of Chinese Academy of Sciences, Beijing 100049, China; State Key Laboratory of Virology, Wuhan Institute of Virology, Center for Biosafety Mega-Science, Chinese Academy of Sciences, Wuhan 430071, China; State Key Laboratory of Drug Research and CAS Key Laboratory of Receptor Research, Shanghai Institute of Materia Medica, Chinese Academy of Sciences, Shanghai 201203, China; School of Pharmaceutical Science and Technology, Hangzhou Institute for Advanced Study, UCAS, Hangzhou 310024, China; School of Chinese Materia Medica, Nanjing University of Chinese Medicine, Nanjing 210023, China; Shanghai Institute for Advanced Immunochemical Studies and School of Life Science and Technology, ShanghaiTech University, Shanghai 201210, China; State Key Laboratory of Drug Research and CAS Key Laboratory of Receptor Research, Shanghai Institute of Materia Medica, Chinese Academy of Sciences, Shanghai 201203, China; University of Chinese Academy of Sciences, Beijing 100049, China; The National Center for Drug Screening, Shanghai Institute of Materia Medica, Chinese Academy of Sciences, Shanghai 201203, China; The National Center for Drug Screening, Shanghai Institute of Materia Medica, Chinese Academy of Sciences, Shanghai 201203, China; School of Pharmaceutical Science and Technology, Hangzhou Institute for Advanced Study, UCAS, Hangzhou 310024, China; State Key Laboratory of Drug Research and CAS Key Laboratory of Receptor Research, Shanghai Institute of Materia Medica, Chinese Academy of Sciences, Shanghai 201203, China; The National Center for Drug Screening, Shanghai Institute of Materia Medica, Chinese Academy of Sciences, Shanghai 201203, China; State Key Laboratory of Drug Research and CAS Key Laboratory of Receptor Research, Shanghai Institute of Materia Medica, Chinese Academy of Sciences, Shanghai 201203, China; The National Center for Drug Screening, Shanghai Institute of Materia Medica, Chinese Academy of Sciences, Shanghai 201203, China; State Key Laboratory of Drug Research and CAS Key Laboratory of Receptor Research, Shanghai Institute of Materia Medica, Chinese Academy of Sciences, Shanghai 201203, China; Shanghai Institute for Advanced Immunochemical Studies and School of Life Science and Technology, ShanghaiTech University, Shanghai 201210, China; The National Center for Drug Screening, Shanghai Institute of Materia Medica, Chinese Academy of Sciences, Shanghai 201203, China; State Key Laboratory of Drug Research and CAS Key Laboratory of Receptor Research, Shanghai Institute of Materia Medica, Chinese Academy of Sciences, Shanghai 201203, China; The National Center for Drug Screening, Shanghai Institute of Materia Medica, Chinese Academy of Sciences, Shanghai 201203, China; State Key Laboratory of Drug Research and CAS Key Laboratory of Receptor Research, Shanghai Institute of Materia Medica, Chinese Academy of Sciences, Shanghai 201203, China; State Key Laboratory of Drug Research and CAS Key Laboratory of Receptor Research, Shanghai Institute of Materia Medica, Chinese Academy of Sciences, Shanghai 201203, China; The National Center for Drug Screening, Shanghai Institute of Materia Medica, Chinese Academy of Sciences, Shanghai 201203, China; State Key Laboratory of Drug Research and CAS Key Laboratory of Receptor Research, Shanghai Institute of Materia Medica, Chinese Academy of Sciences, Shanghai 201203, China; The National Center for Drug Screening, Shanghai Institute of Materia Medica, Chinese Academy of Sciences, Shanghai 201203, China; State Key Laboratory of Drug Research and CAS Key Laboratory of Receptor Research, Shanghai Institute of Materia Medica, Chinese Academy of Sciences, Shanghai 201203, China; State Key Laboratory of Drug Research and CAS Key Laboratory of Receptor Research, Shanghai Institute of Materia Medica, Chinese Academy of Sciences, Shanghai 201203, China; The National Center for Drug Screening, Shanghai Institute of Materia Medica, Chinese Academy of Sciences, Shanghai 201203, China; State Key Laboratory of Drug Research and CAS Key Laboratory of Receptor Research, Shanghai Institute of Materia Medica, Chinese Academy of Sciences, Shanghai 201203, China; University of Chinese Academy of Sciences, Beijing 100049, China; State Key Laboratory of Drug Research and CAS Key Laboratory of Receptor Research, Shanghai Institute of Materia Medica, Chinese Academy of Sciences, Shanghai 201203, China; School of Pharmaceutical Science and Technology, Hangzhou Institute for Advanced Study, UCAS, Hangzhou 310024, China; University of Chinese Academy of Sciences, Beijing 100049, China; State Key Laboratory of Virology, Wuhan Institute of Virology, Center for Biosafety Mega-Science, Chinese Academy of Sciences, Wuhan 430071, China; Shanghai Institute for Advanced Immunochemical Studies and School of Life Science and Technology, ShanghaiTech University, Shanghai 201210, China; State Key Laboratory of Drug Research and CAS Key Laboratory of Receptor Research, Shanghai Institute of Materia Medica, Chinese Academy of Sciences, Shanghai 201203, China; School of Pharmaceutical Science and Technology, Hangzhou Institute for Advanced Study, UCAS, Hangzhou 310024, China; University of Chinese Academy of Sciences, Beijing 100049, China; State Key Laboratory of Drug Research and CAS Key Laboratory of Receptor Research, Shanghai Institute of Materia Medica, Chinese Academy of Sciences, Shanghai 201203, China; School of Pharmaceutical Science and Technology, Hangzhou Institute for Advanced Study, UCAS, Hangzhou 310024, China; University of Chinese Academy of Sciences, Beijing 100049, China; CAS Center for Excellence in Biomacromolecules, Chinese Academy of Sciences, Beijing 100101, China; State Key Laboratory of Virology, Wuhan Institute of Virology, Center for Biosafety Mega-Science, Chinese Academy of Sciences, Wuhan 430071, China; Shanghai Institute for Advanced Immunochemical Studies and School of Life Science and Technology, ShanghaiTech University, Shanghai 201210, China; Shanghai Institute for Advanced Immunochemical Studies and School of Life Science and Technology, ShanghaiTech University, Shanghai 201210, China; State Key Laboratory of Drug Research and CAS Key Laboratory of Receptor Research, Shanghai Institute of Materia Medica, Chinese Academy of Sciences, Shanghai 201203, China; School of Pharmaceutical Science and Technology, Hangzhou Institute for Advanced Study, UCAS, Hangzhou 310024, China; University of Chinese Academy of Sciences, Beijing 100049, China; State Key Laboratory of Virology, Wuhan Institute of Virology, Center for Biosafety Mega-Science, Chinese Academy of Sciences, Wuhan 430071, China; State Key Laboratory of Drug Research and CAS Key Laboratory of Receptor Research, Shanghai Institute of Materia Medica, Chinese Academy of Sciences, Shanghai 201203, China; School of Pharmaceutical Science and Technology, Hangzhou Institute for Advanced Study, UCAS, Hangzhou 310024, China; CAS Center for Excellence in Biomacromolecules, Chinese Academy of Sciences, Beijing 100101, China; Zhongshan Branch, the Institute of Drug Discovery and Development, Chinese Academy of Sciences, Guangdong 528400, China; The National Center for Drug Screening, Shanghai Institute of Materia Medica, Chinese Academy of Sciences, Shanghai 201203, China; State Key Laboratory of Drug Research and CAS Key Laboratory of Receptor Research, Shanghai Institute of Materia Medica, Chinese Academy of Sciences, Shanghai 201203, China; School of Pharmaceutical Science and Technology, Hangzhou Institute for Advanced Study, UCAS, Hangzhou 310024, China; School of Chinese Materia Medica, Nanjing University of Chinese Medicine, Nanjing 210023, China; Zhongshan Branch, the Institute of Drug Discovery and Development, Chinese Academy of Sciences, Guangdong 528400, China

**Keywords:** high-throughput screening, SARS, CoV-2, main, papain-like, proteases

## Abstract

The global COVID-19 coronavirus pandemic has infected over 109 million people, leading to over 2 million deaths up to date and still lacking of effective drugs for patient treatment. Here, we screened about 1.8 million small molecules against the main protease (M^pro^) and papain like protease (PL^pro^), two major proteases in severe acute respiratory syndrome-coronavirus 2 genome, and identified 1851M^pro^ inhibitors and 205 PL^pro^ inhibitors with low nmol/l activity of the best hits. Among these inhibitors, eight small molecules showed dual inhibition effects on both M^pro^ and PL^pro^, exhibiting potential as better candidates for COVID-19 treatment. The best inhibitors of each protease were tested in antiviral assay, with over 40% of M^pro^ inhibitors and over 20% of PL^pro^ inhibitors showing high potency in viral inhibition with low cytotoxicity. The X-ray crystal structure of SARS-CoV-2 M^pro^ in complex with its potent inhibitor 4a was determined at 1.8 Å resolution. Together with docking assays, our results provide a comprehensive resource for future research on anti-SARS-CoV-2 drug development.

## Graphical Abstract

**Graphical Abstract:**
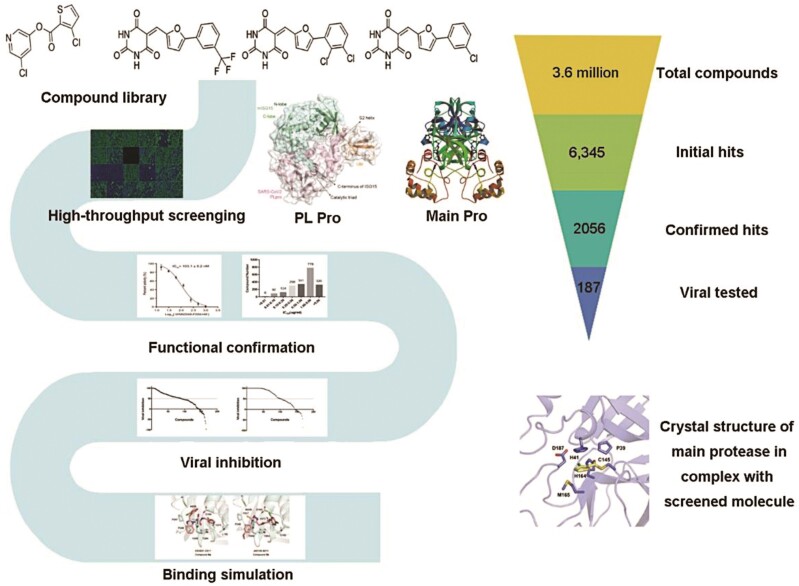

## Introduction

The outbreak of coronavirus disease 2019 (COVID-19) has infected over 109 million cumulative cases with a ~2.2% case-fatality rate globally, and caused worldwide social and economic disruption (Adhikari et al. 2020; Walker et al. 2020). Severe acute respiratory syndrome-coronavirus 2 (SARS-CoV-2) is a positive strand RNA virus that causes severe COVID-19 respiratory disease in human (Guy et al. 2020; Wu et al. 2020b). Several existing drugs that have been applied in clinic to treat COVID-19, such as Lopinavir and Ritonavir, have shown limited curative effect with relatively severe side effects (Cao et al. 2020; Grein et al. 2020b). Remdesivir, an RNA-dependent RNA polymerase (RdRp, EC:2.7.7.48) inhibitor developed for treating Ebola virus (Tchesnokov et al. 2019; Yin et al. 2020), has shown reduced time to clinical recovery, however, more data are still required to confirm its benefits on mild or moderate patients (Durante-Mangoni et al. 2020; Grein et al. 2020a). Currently, no specific anti-SARS-CoV-2 drug is available yet.

Great efforts have been made to characterize molecular targets, which are pivotal for the development of anti-coronaviral therapies. Two of the best-characterized drug targets among coronaviruses are the main protease (M^pro^, also called 3CL^pro^, EC:3.4.22.69) and the papain-like protease (PL^pro^, EC:3.4.22.2), which are responsible for processing the polyproteins pp1a and pp1ab into mature non-structural proteins (Nsps) (Freitas et al. 2020; Wu et al. 2020a). M^pro^ is firstly auto-cleaved from poly-proteins, and then further processes downstream Nsp proteins to release Nsp4–Nsp16, including the RdRp and helicase that are essential in the life cycle of the virus (Lobo-Galo et al. 2020; Zhang et al. 2020). PL^pro^ is responsible for the cleavages of N-terminus of the replicase polyprotein to release Nsp1, Nsp2, and Nsp3 that are required for correcting virus replication (Lin et al. 2018). In addition, PL^pro^ was also confirmed to be significant for antagonizing the host’s immune responses (Ma-Lauer et al. 2016). Thus, both proteases are considered as drug targets for coronaviruses such as SARS-CoV-2, and many efforts had been carried out to identify their novel inhibitors. Several high affinity M^pro^ small molecule inhibitors have already been discovered (Dai et al. 2020b; Jin et al. 2020b), however, no effective PL^pro^ inhibitor of SARS-CoV-2 has been reported yet.

Here, we screened about 1,800,000 compounds from the Chinese National Compound Library (CNCL) for the discovery of inhibitors of M^pro^ and PL^pro^, and identified 1,851 and 205 hits targeting these two proteases, respectively, with great structural diversity. Together with molecular docking, cell-based antiviral assays, and X-ray crystallography, this work provides a systematic framework for further drug development for the treatment of COVID-19.

## Results

### High-throughput screening of M^pro^ inhibitors

**Figure 1.**
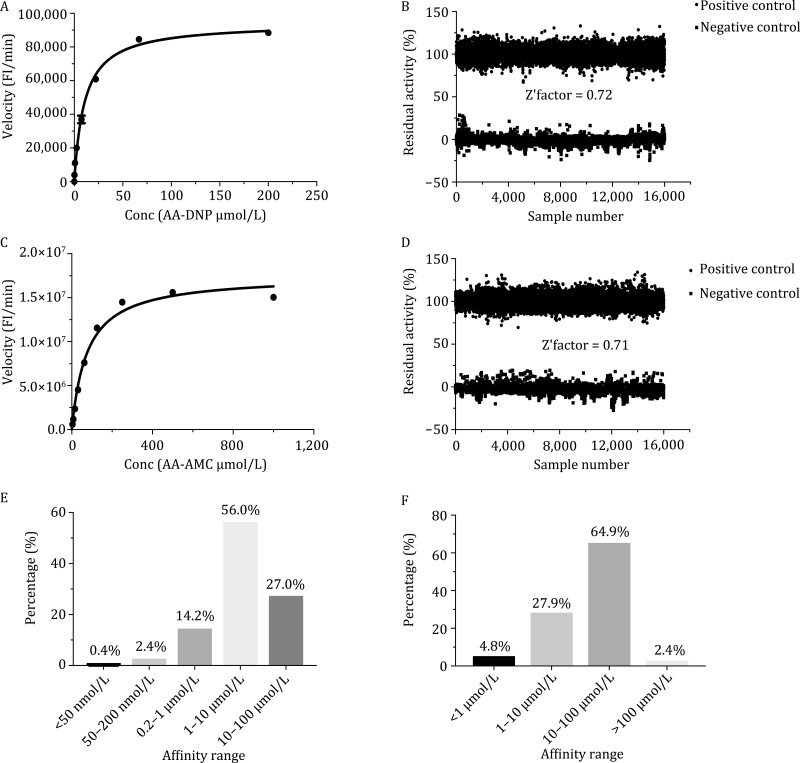
High-throughput screening of M^pro^ and PL^pro^ inhibitors. (A and B) The optimized activity assay (A) and the consistency of high-throughput screening systems (B) targeting on M^pro^ based on about 6000 HTS assay plates. (C and D) The optimized activity assay (C) and the consistency of high-throughput screening systems (D) targeting on PL^pro^ based on 6000 HTS assay plates. (E and F) The IC_50_ distribution of M^pro^ (E) and PL^pro^ (F) inhibitors.

To screen inhibitors, large scale of M^pro^ protein sample with native N and C termini was expressed in *E. coli* as described and purified in a modified protocol (Fig. S1A). The protease activity was tested using the fluorescently labelled substrate MCA-AVLQSGFR-Lys(Dnp)-Lys-NH2. To better facilitate high-throughput screening, the assay system was optimized by screening the best substrate and enzyme concentrations, and the final concentrations were set at 20 µmol/L and 40 nmol/L, respectively, with *Z*ʹ factor of 0.72 (Fig. 1A and 1B). A total 1,733,782 compounds from CNCL were initially screened with the criterion of >70% inhibition at 10 µg/mL. In total, 9,742 compounds were obtained from primary screening with the hit rate of 0.56%. These compounds were further confirmed in a second test with 1 and 10 µg/mL compound concentrations, respectively, and 2358 hits showing inhibition higher than 50% at 1 µg/mL were selected. The half maximal inhibitory concentration (IC_50_) of these compounds was further measured and 1,851 of them exhibit a dose dependent manner with the best IC_50_ of 9.0 ± 4.0 nmol/L, which is the most potent inhibitor reported to date (Dai et al. 2020b) (for detailed chemical structures and affinity data, please visit https://app.cncl.org.cn/). Among these compounds, 0.4% (8 compounds) showed an IC_50_ value below 50 nmol/L, 2.2% (41 compounds) fell in the IC_50_ range of 50–200 nmol/L, 13.7% (253 compounds) showed an IC_50_ value between 200 nmol/L and 1 µmol/L, 56% (1,044 compounds) showed an IC_50_ value of 1–10 µmol/L, and 27% (505 compounds) showed an IC_50_ value between 10 and 100 µmol/L (Fig. 1E; Table S1). The 1,851 compounds were analyzed using cheminformatics method and further classified into over 400 different chemical structures, showing huge diversity of chemical scaffolds and great potential in future drug development.

### High-throughput screening of PL^pro^ inhibitors

Large scale of PL^pro^ protein sample was expressed in *E. coli* and purified in a modified protocol (Fig. S1b). The protease activity was tested using the fluorescently labelled substrate Z-RLRGG-AMC, and the substrate and enzyme concentrations were set at 50 µmol/l and 40 nmol/L, respectively, with the *Z*ʹ factor of 0.71 (Fig. 1C and 1D). The compounds from CNCL were initially screened with a criterion of > 50% inhibition at 8 µg/mL for 1,786,016 compounds and 20 µmol/L for 3,987 bio-active compounds including approved drugs, clinical trial drug candidates, and preclinical drug candidates. 3,987 compounds were obtained and further confirmed in a second test at concentrations of 1 and 10 µg/mL for pure compounds and 2 and 20 µmol/L for bio-active compounds. Overall 387 compounds showing simple dose dependency were selected with more than 50% inhibition against PL^pro^ at 10 µg/mL and 40 bio-active compounds at 20 µmol/L. In total, 205 out of 387 compounds exhibited valid IC_50_ and the best compound showed an IC_50_ value of 0.18 ± 0.03 µmol/L, which is the most potent PL^pro^ inhibitor reported to date (Baez-Santos et al. 2015) (for detailed chemical structures and affinity data, please visit https://app.cncl.org.cn/). Among these compounds, 5.3% (10 compounds) showed an IC_50_ value below 1 µmol/L, 29.0% (59 compounds) fell in the range of 1–10 µmol/L, 62.8% (130 compounds) showed an IC_50_ value between 10 and 100 µmol/L, and 2.9% (6 compounds) showed an IC_50_ value higher than 100 µmol/L (Fig. 1F; Table S2).

Interestingly, among the 205 PL^pro^ inhibitors, six compounds have also shown inhibitory activity in the M^pro^ screening (Table S3). These compounds are classified as 4-phenyl-4,5-dihydro-1H-1,2,4-triazole derivatives. Most of these inhibitors display potency preferences to either protease. For example, compound 3a showed high inhibitory activity against M^pro^ (0.5 ± 0.0 µmol/L) but relatively low inhibitory activity toward PL^pro^ (58.8 ± 15.3 µmol/L). However, another analogue, compound 3b, showed high inhibitory activity against PL^pro^ (6.0 ± 0.0 µmol/L) but low inhibitory activity toward M^pro^ (21.9 ± 0.3 µmol/L). Another derivative, compound 3c, showed a weaker bias between these two proteases with roughly 2-fold difference (4.2 ± 0.6 vs. 10.8 ± 0.5 µmol/L). These results suggest that the development of inhibitors with high potencies toward both M^pro^ and PL^pro^ is possible and our work thus provide attractive hints for developing better anti-SARS-CoV-2 drugs by inhibiting both of its proteases.

### Structure of SARS-CoV-2 M^pro^ in complex with compound 4a

Among the M^pro^ inhibitors with the highest inhibitory activities, 77 compounds showed at least 50% inhibition of viral replication at the concentration of 10 µg/mL (Fig. 3A; Tables S1 and S2). An initial cytotoxicity assays using same inhibitor concentration revealed that only 4.8% (8 out of 166) of M^pro^ inhibitors showed over 30% of cytotoxicity, displaying good compound safety. Among these M^pro^ inhibitors, a compound 4a exhibited high inhibitory potency with IC_50_ value of 0.10 ± 0.05 µmol/L and showed good anti-SARS-CoV-2 infection activity in cell culture with IC_50_ value of 21.3 µmol/L without cytotoxicity effect.

We determined the crystal structure of compound 4a-bound SARS-CoV-2 M^pro^ to elucidate the molecular basis of the compound-induced inhibition of M^pro^. The structure of SARS-CoV-2 M^pro^ contains three domains with the substrate-binding site located in the cleft between domains I and II (Fig. 4C; Table S4). At the active site of SARS-CoV-2 M^pro^, Cys145, and His41 (Cys-His) form a catalytic dyad. The thiol of Cys145 is able to anchor inhibitors by a covalent linkage, which has been reported to be important for the inhibitors to maintain antiviral activity (Yang et al. 2005; Dai et al. 2020b; Jin et al. 2020b). The electron density map showed compound 4a covalently bind to the substrate-binding pocket of SARS-CoV-2 M^pro^ (Fig. 4C). The ester group of compound 4a is employed as a new warhead to form a covalent bond with the Cys145 (Fig. 4D). The thiophene group of compound 4a stacks with the imidazole ring of His41. This group is also surrounded by the side chains of Pro39, His164, Met165, and Asp187. The overall structure of the compound 4a-bound M^pro^ is similar to the previously reported SARS-CoV-2 M^pro^ complex structures (Dai et al. 2020a; Douangamath et al. 2020; Fu et al. 2020; Hoffman et al. 2020; Jin et al. 2020a; Kneller et al. 2020; Sacco et al. 2020; Su et al. 2020; Yang et al. 2020; Zhang et al. 2020; Bai et al. 2021; Lockbaum et al. 2021; Qiao et al. 2021). A major difference lies in the substrate-binding pocket, where the compound 4a has a slightly deeper insertion and induces the outward flip of the His41 to facilitate the ligand binding (Fig. 4C and 4D).

### Binding models of SARS-CoV-2 M^pro^ noncovalent inhibitors

The apparent IC_50_ values of selected inhibitors were measured against a series of different substrate concentrations with *S*/*K*_M_ ratio from 1/2 to 2 (Table S5). Some inhibitors such as compound 4a showed a consistent IC_50_ that is not affected by substrate concentration, indicating a covalent binding manner. The IC_50_ of other inhibitors, such as compound 3a, were decreased as increasing of substrate concentration, suggesting these are noncovalent inhibitors of SARS-CoV-2 M^pro^. To characterize the structure-activity relationships of these M^pro^ inhibitors, molecular docking was performed against the compound 4a bound SARS-CoV-2 M^pro^ and the published structures of SARS-CoV-2 M^pro^ (Dai et al. 2020b; Jin et al. 2020b; Zhang et al. 2020). The inhibitor-bound models were built targeting the substrate-binding site between the domains I and II of M^pro^. 1,471 out of the 1,851 inhibitors could stably bind to the models of M^pro^ in the docking simulations, suggesting that some inhibitors are allosteric modulators or potentially recognize a different conformation of M^pro^. Among the inhibitors that were docked into the M^pro^ models, great chemotype differences were observed and these inhibitors showed distinct binding modes to the protease. Here we present the binding models of several representative chemical scaffolds of inhibitors, which could be starting points for rational development of anti-virus drugs targeting M^pro^.

**Figure 2.**
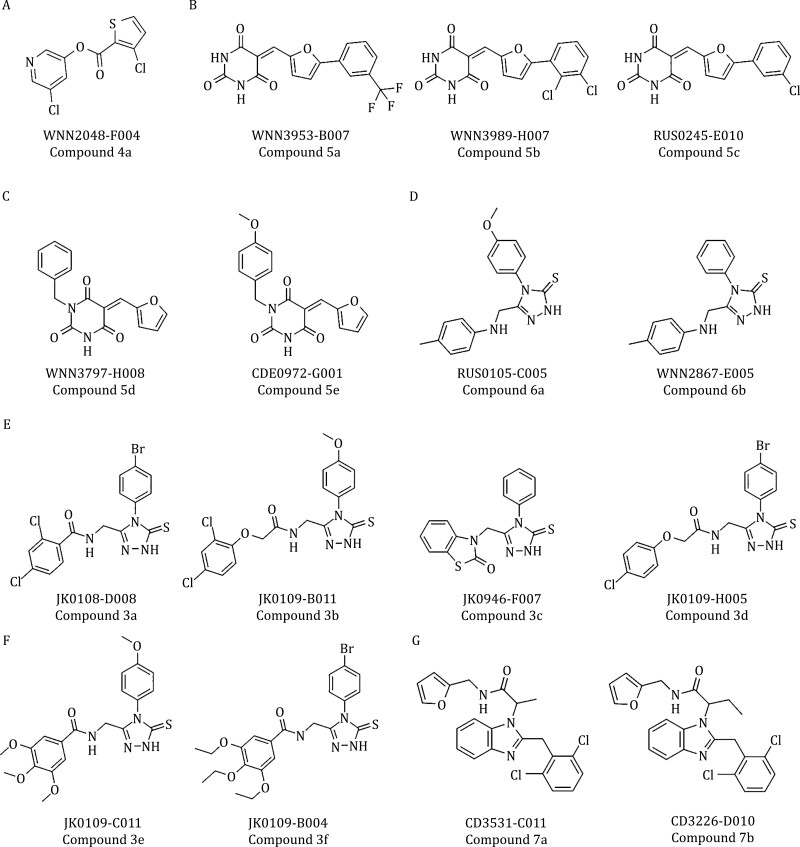
Chemical structures for selected hits targeting SARS-CoV-2 virus proteases. (A) A potent covalent inhibitor of M^pro^. (B–F) Representative M^pro^ inhibitors. (G) PL^pro^ inhibitors with the benzimidazole group.

Five of the potent M^pro^ inhibitors (compounds 5a, 5b, 5c, 5d, and 5e) (Fig. 2B and 2C) have 5-(furan-2-ylmethylene) pyrimidine-2,4,6(1*H*,3*H*,5*H*)-trione group (group R1). In the docking models, this group is located in the center of the substrate-binding site and directly interacts with the Cys-His catalytic dyad (Fig. 5A and 5B). This series of compounds can be further divided into two types: a phenyl group in the furan (compound 5a, 5b, and 5c) and a phenyl group in the pyrimidinone (compounds 5d and 5e). For the furan derived compounds, e.g., compound 5a (Fig. 4A), the phenyl groups (group R2) deeply insert into the hydrophobic cavity consisting of residues Met49, Met165, and Leu176 (Fig. 5A). For the pyrimidinone derived compounds, e.g., compound 5d (Fig. 5B), their phenyl groups (group R3) attach to the edge of the substrate-binding pocket close to the residue Asn142 (Fig. 5B). The phenyl groups of these two types bind to the protein at different subsites, but both are favorable for ligand binding. Since these two types of phenyl-group substitutions are not mutually excluded, it might be possible to develop more specific potent M^pro^ inhibitors based on this scaffold.

**Figure 3.**
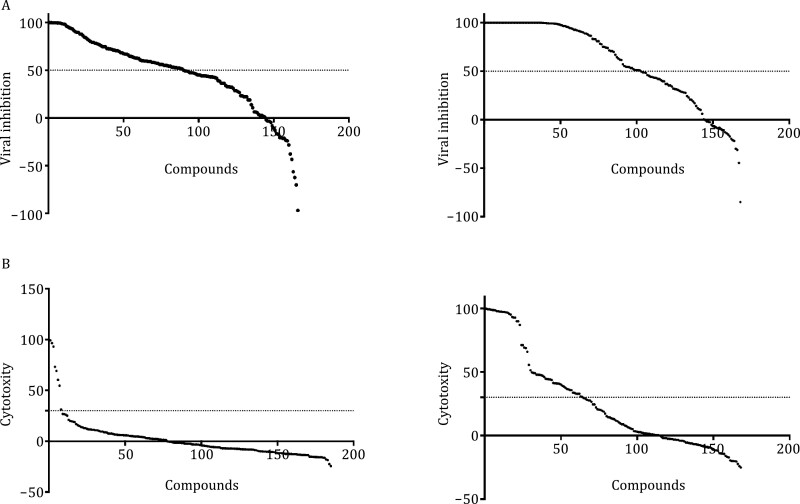
Distribution of virus replication inhibition and cytotoxicity. (A) The inhibition of selected M^pro^ inhibitors (left panel) and PL^pro^ (right panel) on viral replication. (B) The cytotoxicity distribution of selected M^pro^ inhibitors (left panel) and PL^pro^ (right panel) on viral replication. Note that the compound order between (A) and (B) is different.

**Figure 4.**
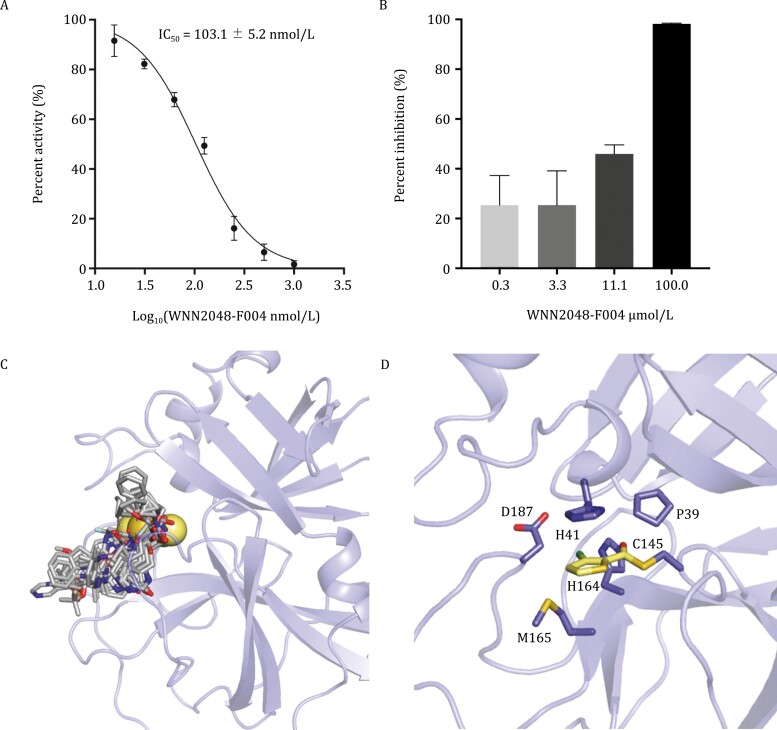
Structure of SARS-CoV-2 M^pro^ in complex with compound 4a. (A) Biochemical inhibition of SARS-CoV-2 M^pro^ by WNN2048-F004. (B) Anti-SARS-CoV-2 infection activity of WNN2048-F004 at different concentrations (0.3, 3.3, 10, and 100 µmol/L). (C) The binding modes of compound 4a and known representative inhibitors with SARS-CoV-2 M^pro^ showed by superimposing all the crystal structures of inhibitor-bound SARS-CoV-2 M^pro^ (PDB codes: 5R84, 6LU7, 6LZE, 6M2N, 6XBH, 6XHM, 6XQT, 6Y2G, 7BQY, 7BRP, 7C7P, 7D1O, 7JPZ, and 7L0D). The compound 4a is shown in yellow spheres. The other inhibitors are shown in gray sticks. (D) The binding pocket of compound 4a. The key residues are shown in sticks and compound 4a is shown in yellow sticks.

**Figure 5.**
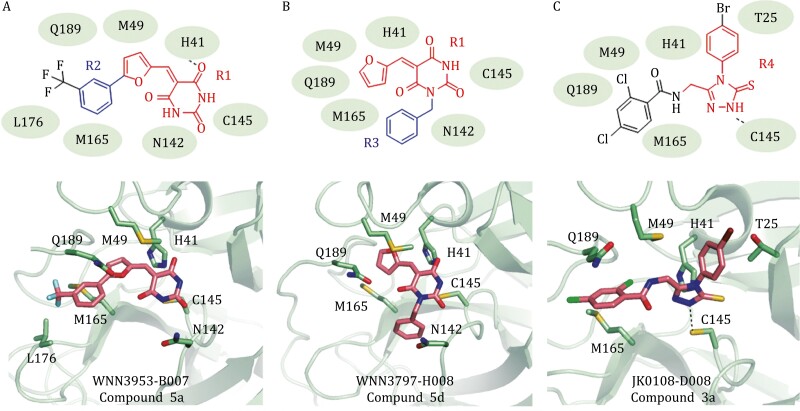
Binding modes of M^pro^ noncovalent inhibitors. (A) Schematic diagram and cartoon representation of the interactions between WNN3953-B007 (compound 5a) and SARS-CoV-2 M^pro^ (PDB code: 6LU7). (B) Schematic diagram and cartoon representation of the interactions between WNN3797-H008 (compound 5d) and SARS-CoV-2 M^pro^ (PDB code: 6LU7). (C) Schematic diagram and cartoon representation of the interactions between JK0108-D008 (compound 3a) and SARS-CoV-2 M^pro^ (PDB code: 6Y2G).

A large series of M^pro^ inhibitors (compounds 3a–3f, 6a, and 6b) (Fig. 2) share a same group of 4-phenyl-2,4-dihydro-3H-1,2,4-triazole-3-thione (group R4). Their 1,2,4-triazole-3-thione group hydrophobically interacts with Thr25 and Met49 and electrostatically interacts with His41 and Cys145 of the catalytic site (Fig. 5C). These compounds have different substituted aromatic groups at the fifth position of the triazole ring. Such aromatic group, including benzo and phenyl, is sandwiched by the hydrophobic residues Met49 and Met165 to facilitate the ligand binding (Fig. 4C). However, some compounds such as compound 3b (Fig. 2E) have long and/or large substituting groups that cannot insert deeply into the substrate-binding pocket. As a result, these compounds showed weak effects on inhibiting M^pro^ (Table S1). Notably, several compounds of these series (compounds 3a–3f) also inhibit PL^pro^.

### Binding models of SARS-CoV-2 PL^pro^ inhibitors

A similar IC_50_ against a series of different substrate concentrations with *S*/*K*_M_ ratio from 1/4 to 4 (Table S6) were also tested on selected SARS-CoV-2 PL^pro^ inhibitors, and similar to SARS-CoV-2 M^pro^, both covalent and noncovalent PL^pro^ inhibitors were suggested. In cell-based assays, 72 out of 121 PL^pro^ inhibitors showed at least 50% inhibition of viral replication at the concentration of 10 µg/mL (Fig. 3A; Tables S1 and S2). An initial cytotoxicity assays using same inhibitor concentration revealed that 38.8% (47 out of 121) of PL^pro^ inhibitors exposed over 30% of cytotoxicity (Fig. 3B). This finding suggested that despite low sequence similarity, PL^pro^ might share a similar binding site with its isozymes in host cells.

We applied molecular docking to characterize the binding modes of the potent PL^pro^ inhibitors. The published structures of SARS-CoV-2 PL^pro^ were used in the molecular docking of PL^pro^ inhibitors. To include more structural information for docking analysis, we also performed molecular docking against homology models generated by using the SARS-CoV PL^pro^ structures as templates (Ratia et al. 2006; Daczkowski et al. 2017). PL^pro^ has an independent ubiquitin-like domain and a right-hand like architecture, including the palm, thumb, and finger domains with its catalytic triad located between the palm and thumb domains. Docking results suggest that instead of direct interactions with the catalytic triad (Ratia et al. 2008), the inhibitors bind to a cleft next to the catalytic site, inducing a loop closure that shuts down catalysis site. Compared with the docking results of M^pro^, a much smaller percentage of the PL^pro^ inhibitors bind to the docking models with high affinity, implying either more accurate models or better understanding of PL^pro^ inhibition mechanism is required.

A series of PL^pro^ inhibitors with the benzimidazole group (compounds 7a and 7b) (Fig. 2G) bind to the allosteric ligand binding site (Freitas et al. 2020). The majority of contacts between PL^pro^ and these compounds are hydrophobic. For example, in the docking model of compound 7a, the benzimidazole group (group R5) of a compound is surrounded by residues Met208, Pro247, Pro248, Tyr268, and Thr301, and its amide group (group R6) forms a hydrogen bond with Asp164 (Fig. 5A). The methyl or ethyl group (group R7) connecting the benzimidazole and amide groups points directly into the interior of the protein between Tyr273 and Thr301. The aromatic group connected to the amide group inserts into the cleft formed by Leu162, Tyr264, and Tyr273, while the other aromatic group connected to the benzimidazole attaches to the edge of the cleft (Fig. 5A).

**Figure 6.**
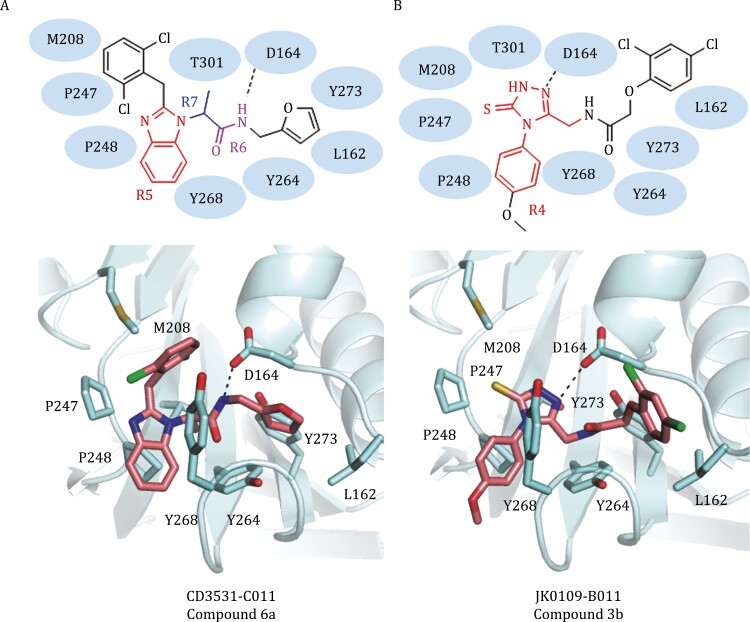
Binding modes of PLpro inhibitors. (A) Schematic diagram and cartoon representation of the interactions between CD3531-C011 (compound 6a) and SARS-CoV-2 PLpro (template PDB code: 3MJ5). (B) Schematic diagram and cartoon representation of the interactions between JK0109-B011 (compound 3b) and SARS-CoV-2 PLpro (template PDB code: 3E9S).

Several M^pro^ inhibitors also diminish the activity of PL^pro^. In the docking models of PL^pro^, the 4-phenyl-2,4-dihydro-3*H*-1,2,4-triazole-3-thione compound series (compounds 3a–3f) had their 4-phenyl-2,4-dihydro-3*H*-1,2,4-triazole-3-thione group (R4) insert into the allosteric ligand-binding site formed by Asp164, Met208, Pro247, Pro248, and Tyr268 (Fig. 6B). Asp164 is highly conserved among most coronaviral papain-like proteases and has been revealed to be important for ligand stabilization (Sulea et al. 2006; Ratia et al. 2008). For these compounds (compounds 3a–3f), the nitrogen within the triazole group electrostatically interacts with the side chain carboxyl group of the residue Asp164. The substitution at the fifth position of the triazole ring is sandwiched by three tyrosine residues Tyr264, Tyr268, and Tyr273. In general, the PL^pro^ inhibitors contain hydrophobic aromatic rings connected by polar groups, which form hydrogen bonds with the key residue Asp164.

## Discussion

SARS-CoV-2 virus poses a continued threat and has a potential for a new global pandemic. The preparation of drug reserve with diverse compounds not only offers necessary treatments for the infected patients but also helps us quick respond to other viral outbreaks. In this work, we tested approximately 1.8 million small molecules against two major proteases in SARS-CoV-2 genome, i.e., M^pro^ and PL^pro^, and identified 1,851 M^pro^ inhibitors and 205 PL^pro^ inhibitors with low nano-molar activity. 77 M^pro^ inhibitors and 72PL^pro^ inhibitors showed at least 50% inhibition of viral replication at the concentration of 10 µg/mL (Fig. 3A; Tables S1 and S2). Diverse chemotypes were identified in the large number of potent inhibitors, which provide valuable information for the development of small-molecule drug reserve against SARS-CoV-2 virus.

The substrate specificity of M^pro^ is highly conserved among different coronavirus (Fig. S2), making it an ideal target for the development of broad-spectrum antiviral drugs (Yang et al. 2005; Pillaiyar et al. 2016). Several types of substrate-like peptidomimetic M^pro^ inhibitors targeting the substrate-binding site have been reported (Pillaiyar et al. 2016; Zumla et al. 2016; Dai et al. 2020b; Jin et al. 2020b). However, the classical small-molecule covalent inhibitors that are validated by complex structure determination have not emerged. Integrating enzymatic assays with X-ray protein crystallography, we identified compound 4a as the first class covalent, nonpeptidomimetic inhibitor of SARS-CoV-2 M^pro^. The crystal structure reveals the binding mode of compound 4a is different from those of known M^pro^ inhibitors. To resemble the binding of the substrates, the previously reported peptidomimetic inhibitors bind to the substrate-binding pocket in extended delineated conformations and occupy most subsites of the pocket. As a small molecule, compound 4a only occupies one subsite of the catalytic dyad by interacting with His41 and Cys145, which are key elements for the recognition of substrates (Tan et al. 2005). It forms a covalent bond with the Cys145 and stacks with the side chain of His by the thiophene group. Even though the compound 4a only block the catalytic dyad, it is able to effectively prevent the access of substrate to the core of the active site. The unique binding mode and the high inhibitory efficiency make compound 4a a potential lead for future drug development targeting M^pro^. In addition to the covalent inhibitor, we also identified a large number of noncovalent small-molecule inhibitors of M^pro^. Multiple chemical series of M^pro^ inhibitors are identified and distinct binding modes of these inhibitors are characterized using molecular docking. Such findings provide valuable information for developing noncovalent inhibitors targeting M^pro^.

PL^pro^ is also an attractive antiviral drug target. This protease regulates the process of virus protein maturation (Baez-Santos et al. 2015), and participate in inhibiting the production of cytokines and chemokines, which are crucial in the host innate immune response against viral infection (Devaraj et al. 2007; Frieman et al. 2009; Clementz et al. 2010; Calistri et al. 2014; Mielech et al. 2014). Therefore, knowledge of PL^pro^ inhibitors is important for the rational design of antivirus drugs. In this work, we identified 205 potent PL^pro^ inhibitors with diverse chemical structures. 72 PL^pro^ inhibitors showed at least 50% inhibition of viral replication at the concentration of 10 µg/mL (Fig. 3A; Tables S1 and S2), indicating PL^pro^ is a viable target for developing antiviral drugs against SARS-CoV-2. The cytotoxicity assays revealed that 40 PL^pro^ inhibitors exposed over 30% of cytotoxicity (Fig. 3B), suggesting PL^pro^ may share a similar binding site with its isozymes in host cells. SARS-CoV-2 PL^pro^ is homologous to human deubiquitinating enzymes including more than 40 cysteine proteases (Daviet and Colland 2008). The high cytotoxicity of some PL^pro^ inhibitors might be a consequence of the off-target effects on the human deubiquitinating enzymes. In this work, we identified a number of potent PL^pro^ inhibitors with low cytotoxicity. These inhibitors can be directly used in the development of safe drugs targeting pathogenic PL^pro^ without inhibitor host deubiquitinating enzymes.

In short, we performed high-throughput screening of approximately 1.8 million small-molecule compounds against two major proteases, M^pro^ and PL^pro^ of SARS-CoV-2. 2,050 hits were discovered, including six compounds that could inhibit both proteases. Further cell-based antiviral assays and molecular docking results identifies over 100 inhibitors with high antiviral potency as well as low cytotoxicity. A potent covalent inhibitor of M^pro^ was identified through these assays. The X-ray crystal structure of SARS-CoV-2 M^pro^ in complex with its potent inhibitor 4a was determined at 1.8 Å resolution. The compounds identified through our screening paradigm and the molecular basis of the protease-inhibitor interactions revealed in this work will greatly facilitate the future drug development targeting COVID-19 and other coronaviruses.

## Materials and methods

### Expression and purification of M^pro^

The full-length gene encoding SARS-CoV-2M^pro^ protein was code optimized and synthesized in pGEX6p-1vector for *E. coli* expression (Genewiz). The expression plasmid was transformed into *E. coli* BL21 (DE3) cells and then cultured in Luria broth (LB) media containing 100 µg/mL ampicillin at 37 °C, 200 rpm. When the cells were grown to OD_600_ of 0.6–0.8, 0.5 mmol/L IPTG was added to the cell culture to induce the expression of the recombinant 2019-nCoV M^pro^ protein at 16 °C, 180 rpm, overnight, then the cells were harvested by centrifugation at 3000 ×*g* for 20 min. Cell pellets were resuspended in lysis buffer (20 mmol/L Tris–HCl, pH 8.0, 300 mmol/L NaCl), lysed by 4–5 rounds of high-pressure homogenization, and then centrifuged at 25,000 ×*g* for 40 min. The supernatant was loaded onto an Ni-NTA affinity column and binding for 2 h at 4 °C, and washed by gradient concentration of resuspension buffer containing 0–30 mmol/L imidazole. The His tagged M^pro^ protein was eluted by cleavage buffer (50 mmol/L Tris–HCl, pH 7.5, 150 mmol/L NaCl) containing 300 mmol/L imidazole. The sample was treated overnight with His-tagged PreScission protease to remove the C-terminal His tag. The Ni-NTA resin (Qiagen) was incubated with the sample at 4 °C for 1 h to remove the cleaved His-tag and PreScission protease. The purified M^pro^ protein was concentrated using a 10 kDa molecule weight cut-off concentrator (Millipore), and subjected to size exclusion chromatography Superdex 200 increase 10/300 for buffer exchange to 50 mmol/L Tris–HCl, pH 7.3, 1 mmol/L EDTA.

### Expression and purification of PL^pro^

The PL^pro^ was inserted into pET-22b (+) followed by a PreScission protease site and a 6× His-tag at the C terminus. The transformed *E. coli* BL21 (DE3) cells were cultured in LB medium containing 75 µg/mL ampicillin at 37 °C for 4 h. Protein was then induced by adding 0.5 mmol/L IPTG and incubated over-night at 16 °C. The cells were harvested by centrifugation at 6,200 ×*g* for 15 min at 4 °C.

The cell pellet was suspended in binding buffer (50 mmol/L Tris–HCl pH 8.0, 150 mmol/L NaCl, 2 mmol/L DTT) followed by dounce homogenization and cells were disrupted by ultra-high pressure cell disrupters (JNBIO) at 4 °C. After centrifugation at 160,000 ×*g* for 30 min, the supernatant was collected and incubated with Ni-NTA resin supplied with 5 mmol/L imidazole. After a 2 h-incubation at 4 °C, the resin was washed with 30 column volumes of washing buffer I (50 mmol/L Tris–HCl pH 8.0, 150 mmol/L NaCl, 10 mmol/L imidazole, 2 mmol/L DTT) followed by four column volumes of washing buffer II (50 mmol/L Tris–HCl pH 8.0, 150 mmol/L NaCl, 20 mmol/L imidazole, 2 mmol/L DTT) and three column volumes of washing buffer III (50 mmol/L Tris–HCl pH 8.0, 150 mmol/L NaCl, 30 mmol/L imidazole, 2 mmol/L DTT). Then the protein was eluted with elution buffer (50 mmol/L Tris–HCl pH 8.0, 150 mmol/L NaCl, 300 mmol/L imidazole, 2 mmol/L DTT) and further purified by gel filtration using a Superdex 75 (GE Healthcare) gel filtration column.

### Enzymatic activity and inhibition assays

The enzyme activity and inhibition assays of SARS-CoV-2 M^pro^ have been described previously (Dai et al. 2020b; Jin et al. 2020b). Briefly, the recombinant SARS-CoV-2 M^pro^ (40 nmol/L at a final concentration) was mixed with each compound in 50 µL assay buffer (20 mmol/L Tris, pH7.3, 150 mmol/L NaCl, 1 mmol/L EDTA, 1% Glycerol, 0.01% Tween-20) and incubated for 10 min. The reaction was initiated by adding the fluorogenic substrate MCA-AVLQSGFRK (DNP) K (GL Biochem, Shanghai), with a final concentration of 20 µmol/L. After that, the fluorescence signal at 320 nm (excitation)/405 nm (emission) was immediately measured by continuous 8 points for 8 min with an EnVision multimode plate reader (Perkin Elmer, USA). The initial velocity was measured when the protease reaction was proceeding in a linear fashion.

The activity of SARS-CoV-2 PL^pro^ was also measured by a continuous 8 points fluorometric assay for 8 min. Briefly, the recombinant SARS-CoV-2 PL^pro^ (40 nmol/L at a final concentration) was mixed with each compound in 50 µL assay buffer (20 mmol/L Tris pH 8.0, 0.01% Tween20, 0.5 mmol/L DTT) and incubated for 10 min. The reaction was initiated by adding the substrate Z-RLRGG-AMC (GL Biochem, Shanghai) with a final concentration of 50 µmol/L, using wavelengths of 355 nm and 460 nm for excitation and emission, measured by an EnVision multimode plate reader (Perkin Elmer, USA).

### High-throughput screen and IC_50_ measurement

Potential inhibitors against SARS-CoV-2 M^pro^ and PL^pro^ were screened by an enzymatic inhibition assay carried outinblack 384-well plates (OptiPlateTM-384F, PerkinElmer). The *Vmax* of reactions added with different compounds compared to the reaction added with DMSO were calculated and used to generate inhibitory rate and IC_50_. The ~1,800,000 compounds of CNCL bought from ChemDiv (USA), ChemBridge (USA), Life Chemicals (Canada), Specs (Holland) and donated by NovoNordisk (Denmark) were primarily screened against M^pro^ and PL^pro^ at the concentration of 10 and 8 µg/mL respectively. And 3,987 bio-active compounds containing approved drugs, clinical trial drug candidates, preclinical drug candidates bought from MCE (USA), ApeBio (USA), Selleck (USA), and TargetMol (USA) were also primarily screened against PL^pro^ at the concentration of 20 µmol/L. Hits identified from the primary screen assayed in single well were subsequently secondary screened against SARS-CoV-2 M^pro^ and PL^pro^with two concentrations (1 and 10 µg/mL) in duplicates. For selected potential inhibitors which showed two doses dependence and more than 50% inhibition at higher concentration, IC_50_ values against SARS-CoV-2 M^pro^ and PL^pro^ were measured at eight concentrations and three independent experiments were performed. All experimental data was analyzed using GraphPad Prism software.

### Compounds clustering

The original set with 1,873 compounds was further processed by removing the molecules having poorly specified and then represented as Extended-Connectivity Fingerprints1 (ECFP) and tanimoto similarity matrix was calculated. Hierarchical clustering algorithms2 and average linking method were chosen for compounds clustering. Different similarity threshold was tried, and similarity threshold of 0.1 was chosen by clustering performance and synthetic feasibility. Finally, representative structures of 421 group were kept for further use.

### Antiviral assays

Vero cells were seeded in 96-well plates at a density of 6,000 cells per well in a total volume of 100 µL per well and incubated overnight at 37 °C and 5% CO_2_. Cell monolayers were treated with the compounds at a final concentration of 10 µg/mL or 10 µmol/L for 1 h, and infected with SARS-CoV-2 at an MOI of 0.01. At 24 h p.i., cells were fixed and incubated with rabbit anti- NP antibody, followed by anti-rabbit Alexa488 (Abcam) and DAPI (Beyotime). The plates were imaged using Operetta (PerkinElmer) with a 10× objective. Nine images were acquired per well in both the DAPI and 488 nm channels. The percentages of infected and DAPI-positive cells were calculated using automated image analysis software (Harmony 3.5, PerkinElmer). A positive control of anti-SARS-CoV-2 compound (Chloroquine diphosphate salt, Sigma, C6628) and vehicle (DMSO) were handled in the same way as for the compound library during drug screening in a blinded fashion, with the compound identities unknown to the experimenters.

### Homology modeling and molecular docking

Eight published SARS-CoV-2M^pro^ structures deposited in the Protein Data Bank (www.rcsb.org) were selected for molecular docking. Their PDB codes are 6LU7, 6LZE, 6M0K, 6Y2E, 6Y2F, 6Y2G, 7BQY, 7BUY. Six published SARS-CoV-2 PL^pro^ structures deposited in the Protein Data Bank were selected for molecular docking. Their PDB codes are 6W9C, 6WRH, 6YVA, 6WX4, 6WZU, 6WUU. To include more structural information for docking analysis, we also used the ligand-bound SARS-CoV PL^pro^ structures as templates to build the homology models of SARS-CoV-2 PL^pro^ and performed molecular docking against these models. Their PDB codes are 3E9S, 3MJ5, 4M0W, 4OVZ, 4OW0, 5E6J, and 5Y3E. Modeller (Sali 1995) was used to perform homology modeling with the default parameters. Thirteen resulting models with the lowest RMSD from their templates were selected for further analysis. A compound of interest was docked to its receptor using Schrodinger Glide software in SP mode with default parameters (Friesner et al. 2004). The ligand was initially placed in the center of the pocket and was constrained to move within 1 nm diameter sphere, where it was allow freely moving during the docking process. The extended conformation searches were performed using Lamarckian Genetic Algorithm. The docking model with the lowest binding energy was selected for analysis. When the binding energy score of a compound to a receptor model is larger than −6.0, the resulting docking model is excluded.

## Supplementary Material

pwac016_suppl_Supplementary_Materials

## Abbreviations

3CL^pro^: 3C-like protease
CNCL: the Chinese National Compound Library
COVID-19: coronavirus disease 2019
IC_50_: half maximal inhibitory concentration
M^pro^: main protease
PL^pro^: papain like protease
Nsp: non-structural protein
HTS: high-throughput screening
RdRp: RNA-dependent RNA polymerase
SARS-CoV-2: Severe acute respiratory syndrome-coronavirus 2
