# Supplementary material for: High-throughput screening of SARS-CoV-2 main and papain-like protease inhibitors
Source: Protein Cell. 2022 Sep 28;14(1):17–27. doi: 10.1093/procel/pwac016 (PMC9871955; doi:10.1093/procel/pwac016)
Supplement: pwac016_suppl_Supplementary_Materials [file pwac016_suppl_supplementary_materials.docx]

**Supplementary Materials**


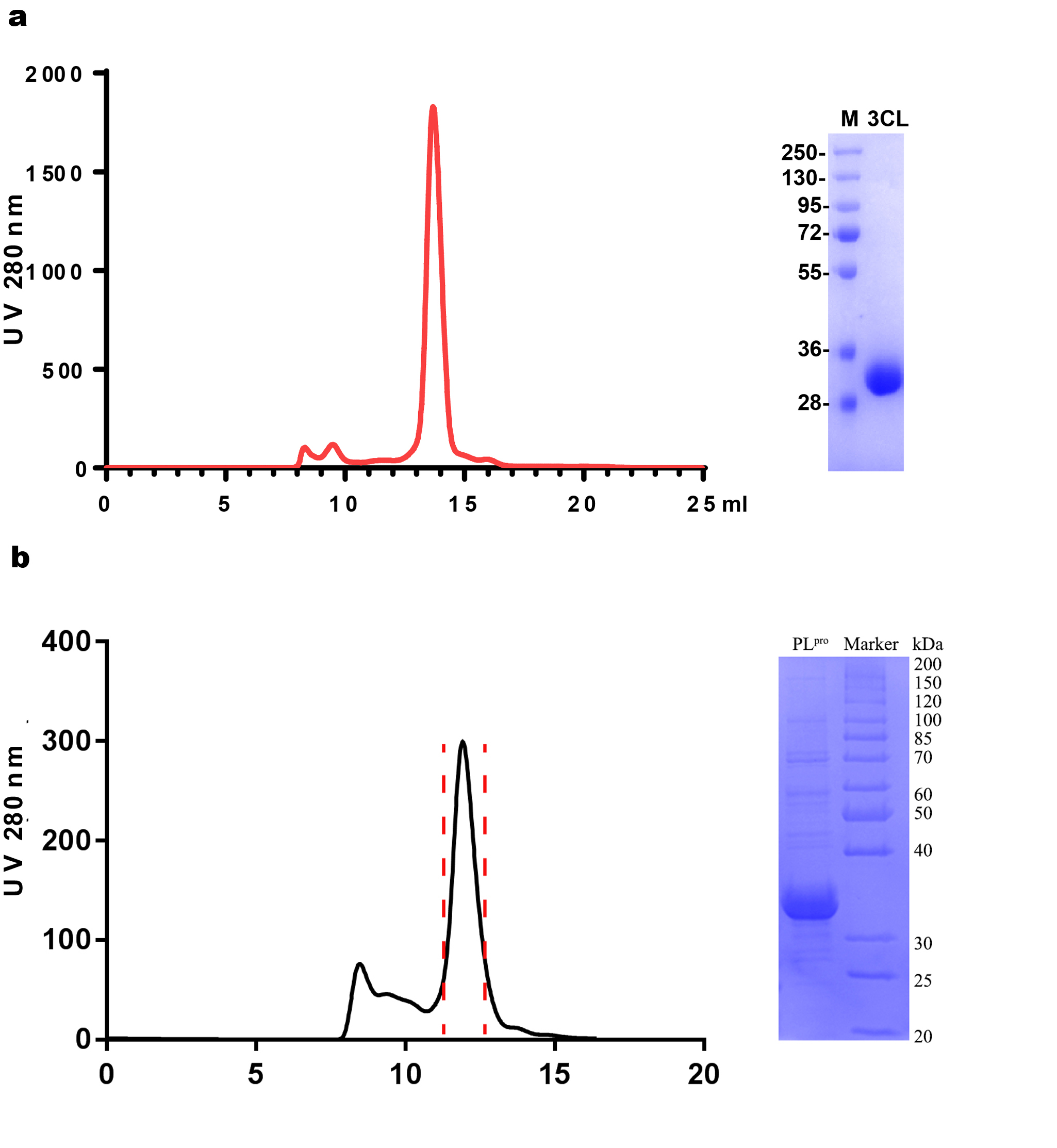


**Figure S1. Purification of M^pro^ and PL^pro^.** a) SEC results and gel purification of M^pro^. b) SEC results and gel purification of PL^pro^.


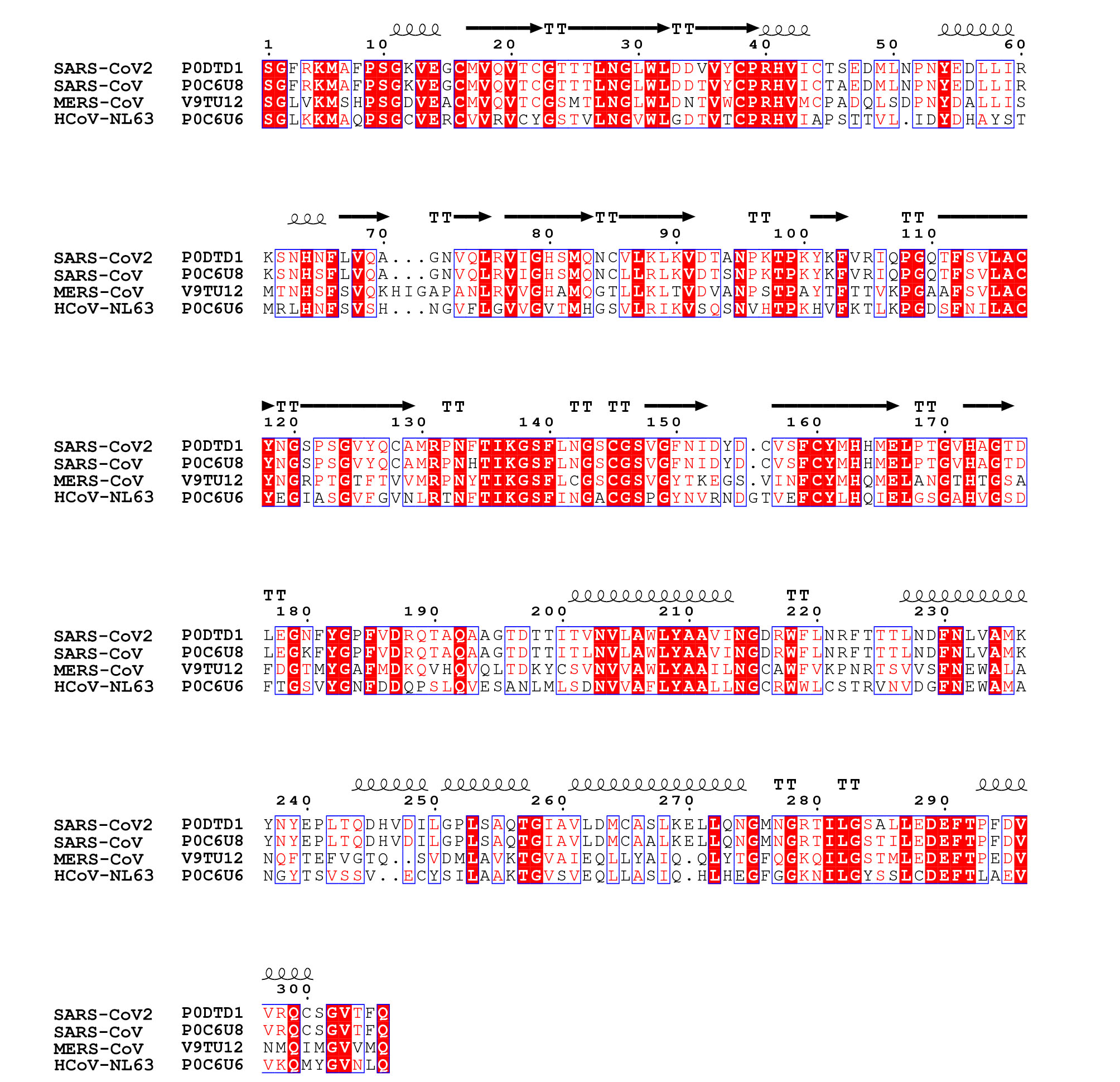


**Figure S2. Sequence alignment of M^pro^ proteins from coronaviruses.** The residue numbering is based on SARS-CoV2 M^pro^. The secondary structure predicted by the dictionary of protein secondary structure is shown for SARS-CoV2 M^pro^. The identical or conserved residues are highlighted in red.

**Table S1.** Summary of IC_50_, antiviral potencies and cytotoxicities of SARS-CoV-2 M^pro^ inhibitors.

| No | Compound | IC_50_ (μM) | Antiviral activity (%) | Cytotoxicity activity (%) |
| --- | --- | --- | --- | --- |
| 1 | NCRW0190-D011 | 0.009±0.004 | 13.84 | -15.49 |
| 2 | WNN3668-H011 | 0.018±0 | -43.36 | -8.62 |
| 3 | WNN2927-G009 | 0.026±0.003 | 51.65 | 5.32 |
| 4 | WNN2678-D008 | 0.032±0.004 | 87.95 | -3.1 |
| 5 | NCRW0342-A002 | 0.043±0 | 2.61 | -6.66 |
| 6 | WNN3697-G005 | 0.044±0 | 52.34 | -7.18 |
| 7 | NCDS0225-C010 | 0.054±0.004 | 83.92 | -9.81 |
| 8 | WNN2349-F007 | 0.055±0.003 | N.D. | 10.81 |
| 9 | WNN3930-A003 | 0.059±0 | 86.05 | -11.16 |
| 10 | WNN0029-C005 | 0.063±0.006 | 99.57 | 93.07 |
| 11 | WNN0300-C003 | 0.065±0.014 | 32.4 | 10.07 |
| 12 | WNN2341-A008 | 0.069±0.003 | N.D. | 6.61 |
| 13 | WNN0202-B006 | 0.078±0 | 32.83 | 16.89 |
| 14 | WNN3206-D010 | 0.086±0.008 | -56.45 | -2.44 |
| 15 | WNN0004-A011 | 0.094±0.003 | 47.49 | 15.12 |
| 16 | RD0091-C009 | 0.113±0.012 | 98.69 | -11.46 |
| 17 | WNN3206-D009 | 0.121±0.016 | 98.42 | -12.72 |
| 18 | WNN3833-G011 | 0.123±0.004 | 82.56 | 19.7 |
| 19 | WNN3927-E011 | 0.124±0.013 | 81.36 | -12.44 |
| 20 | WNN3367-C004 | 0.126±0.022 | 36.46 | -5.04 |
| 21 | WNN3823-B005 | 0.127±0.011 | 36.39 | -10.23 |
| 22 | RD0012-D003 | 0.13±0.015 | 43.97 | -4.02 |
| 23 | WNN0434-E008 | 0.13±0.016 | 42.93 | -11.4 |
| 24 | WNN2048-F004 | 0.131±0.011 | N.D. | 6.16 |
| 25 | WNN3206-D011 | 0.135±0.015 | 22.26 | 1.99 |
| 26 | RUS0770-H004 | 0.14±0.026 | 70.46 | 26.58 |
| 27 | RD0928-B006 | 0.148±0.004 | 44.03 | 73.15 |
| 28 | WNN3625-B003 | 0.149±0.021 | 87.9 | 2.38 |
| 29 | WNN2374-G002 | 0.151±0.021 | N.D. | 2.39 |
| 30 | WNN0466-F004 | 0.153±0.002 | 42.24 | -1.82 |
| 31 | WNN3843-A002 | 0.153±0.012 | -16.54 | -10.78 |
| 32 | WNN3953-B007 | 0.16±0.009 | 79.24 | 13.01 |
| 33 | WNN0529-G007 | 0.162±0.019 | 23.12 | -7.11 |
| 34 | WNN2222-B007 | 0.165±0.004 | N.D. | 11.75 |
| 35 | WNN3797-H008 | 0.172±0 | 59.58 | -7.75 |
| 36 | WNN3765-G007 | 0.175±0.025 | 91.63 | -3.19 |
| 37 | WNN5263-G003 | 0.179±0.002 | -70.26 | 18.86 |
| 38 | WNN3374-E004 | 0.182±0.003 | 74.65 | -7.82 |
| 39 | WNN2945-B002 | 0.185±0.004 | 46.81 | 11.02 |
| 40 | WNN0454-D008 | 0.188±0 | 57.42 | -10.42 |
| 41 | RUS0245-E010 | 0.196±0.009 | 49.93 | -8.14 |
| 42 | WNN3681-A006 | 0.197±0.007 | 18.98 | -2.5 |
| 43 | WNN3985-B011 | 0.201±0.015 | 43.64 | -7.1 |
| 44 | WNN0434-E007 | 0.202±0.013 | 74.7 | -7.03 |
| 45 | JK1173-B005 | 0.211±0.006 | -1.82 | 2.51 |
| 46 | WNN3671-H005 | 0.227±0.008 | 47.48 | -7.39 |
| 47 | WNN2210-C004 | 0.227±0.016 | N.D. | 19.23 |
| 48 | WNN3815-F007 | 0.233±0.102 | 84.64 | -8.07 |
| 49 | JK1163-F003 | 0.239±0.021 | 71.94 | 13.8 |
| 50 | WNN0812-D002 | 0.265±0.011 | 72.42 | 6.36 |
| 51 | WNN2606-F010 | 0.266±0.025 | N.D. | 3.1 |
| 52 | CDE0972-G011 | 0.267±0.006 | 1.78 | -7.84 |
| 53 | WNN2773-H002 | 0.267±0.037 | 58.99 | -12.19 |
| 54 | RD0497-H004 | 0.267±0.079 | 62.55 | -16.25 |
| 55 | RUS0009-E011 | 0.268±0.004 | 58.16 | -17.9 |
| 56 | WNN0411-G007 | 0.275±0.058 | 43.84 | 11.43 |
| 57 | WNN2979-E005 | 0.283±0.015 | 98.1 | 69.09 |
| 58 | NCDS0218-F002 | 0.296±0.044 | 26.84 | -11.43 |
| 59 | WNN0385-C009 | 0.299±0.031 | 18.98 | -2.29 |
| 60 | WNN3172-G003 | 0.304±0.033 | 52.82 | -0.95 |
| 61 | WNN3599-G006 | 0.311±0.003 | 3.53 | -7.98 |
| 62 | NCDS0240-D009 | 0.318±0 | 45.21 | 4.73 |
| 63 | WNN3481-H008 | 0.318±0.071 | -28.31 | -3.7 |
| 64 | WNN3741-B002 | 0.319±0.003 | 72.51 | -10.09 |
| 65 | RUS0433-C010 | 0.319±0.016 | 49.16 | -9.29 |
| 66 | WNN5241-F011 | 0.321±0.032 | 79.16 | -13.48 |
| 67 | WNN3875-A004 | 0.323±0.008 | 10.71 | 3.84 |
| 68 | RUS0910-G009 | 0.324±0.064 | 99.51 | 31.15 |
| 69 | NCRW0355-A008 | 0.326±0.04 | 64.68 | 6.18 |
| 70 | JK0549-B004 | 0.336±0.006 | 58.22 | -8.1 |
| 71 | JK1161-F007 | 0.336±0.028 | 2.64 | 7.53 |
| 72 | JK2114-F005 | 0.342±0.047 | 63.24 | -7.94 |
| 73 | WNN3365-C008 | 0.352±0 | -21.29 | -3.19 |
| 74 | WNN3362-E010 | 0.352±0.003 | 99.55 | 54.57 |
| 75 | WNN3955-A002 | 0.358±0.01 | 53.36 | 4.28 |
| 76 | JK1173-E003 | 0.36±0.004 | -62.41 | 2.33 |
| 77 | CDE0286-D005 | 0.361±0.049 | 38.46 | 7.62 |
| 78 | RUS0360-A008 | 0.365±0.192 | 54.79 | -2.31 |
| 79 | CD0501-D011 | 0.372±0.012 | 99.83 | 96.39 |
| 80 | WNN3952-F009 | 0.372±0.034 | 53.2 | -1.67 |
| 81 | WNN1979-H003 | 0.375±0.013 | N.D. | 21.13 |
| 82 | WNN3726-B006 | 0.379±0.02 | 90.28 | 19.99 |
| 83 | WNN0464-F002 | 0.381±0.07 | 64.36 | -13.03 |
| 84 | CDE0025-H006 | 0.387±0.003 | 36.4 | -10.71 |
| 85 | WNN2809-B002 | 0.389±0.05 | N.D. | -1.51 |
| 86 | WNN3395-G005 | 0.389±0.091 | 48.53 | 12.34 |
| 87 | WNN3273-D002 | 0.394±0.049 | -24.03 | 5.69 |
| 88 | WNN0385-F004 | 0.401±0.016 | 52.76 | 6.73 |
| 89 | CDE1324-C007 | 0.402±0.024 | 66.97 | -6.85 |
| 90 | JK1530-G010 | 0.406±0.058 | -3.56 | -13.55 |
| 91 | WNN1087-E003 | 0.408±0.038 | 66.92 | 11.79 |
| 92 | WNN3445-G003 | 0.418±0.021 | -3.34 | -6.86 |
| 93 | JK1172-G009 | 0.422±0.025 | -4.62 | -15.31 |
| 94 | WNN3650-F005 | 0.425±0.009 | 68.36 | -9.89 |
| 95 | JK3335-G008 | 0.425±0.04 | 22.32 | 7.5 |
| 96 | WNN3926-B011 | 0.435±0.055 | 71.11 | -12.47 |
| 97 | WNN3697-G004 | 0.437±0.003 | 28.76 | 8.53 |
| 98 | WNN3598-A008 | 0.437±0.023 | -37.81 | 6.12 |
| 99 | WNN0454-D007 | 0.441±0.019 | 44.71 | 7.14 |
| 100 | RUS0205-F005 | 0.447±0.113 | 49.28 | -1.47 |
| 101 | WNN1544-B005 | 0.454±0.039 | 68.41 | 1.24 |
| 102 | CB0944-E008 | 0.455±0.06 | 41.55 | -4.22 |
| 103 | WNN3395-H010 | 0.458±0.12 | 80.03 | -5.83 |
| 104 | WNN2867-E005 | 0.466±0.101 | 46.28 | -6.94 |
| 105 | WNN2891-C011 | 0.477±0.027 | 44.05 | 3.03 |
| 106 | RUS0105-C005 | 0.478±0.009 | 28.69 | 5.87 |
| 107 | JK1173-A005 | 0.481±0.178 | -10.93 | 4.29 |
| 108 | JK0944-G009 | 0.488±0.051 | -20.65 | 14.16 |
| 109 | JK2150-C006 | 0.49±0.087 | 99.87 | 60.41 |
| 110 | WNN2898-C010 | 0.492±0 | 59.65 | 9.63 |
| 111 | WNN1075-B010 | 0.496±0.007 | 56.12 | 4.75 |
| 112 | WNN3651-B011 | 0.496±0.067 | 95.4 | -24.39 |
| 113 | WNN2191-E007 | 0.504±0.034 | N.D. | 8.72 |
| 114 | WNN3799-D005 | 0.515±0.011 | 94.5 | 26.11 |
| 115 | NCRW0288-G006 | 0.515±0.018 | 66.05 | -15.59 |
| 116 | NCRW0242-D004 | 0.515±0.332 | 6.44 | -12.72 |
| 117 | NCRW0066-C009 | 0.519±0.014 | 59.93 | -13.58 |
| 118 | WNN3978-B011 | 0.523±0.003 | 44.93 | -10.05 |
| 119 | CD0267-D007 | 0.525±0.064 | 60.19 | -4.44 |
| 120 | WNN3673-F011 | 0.527±0.054 | 34.03 | -5.96 |
| 121 | WNN2412-H006 | 0.535±0.071 | N.D. | -1.41 |
| 122 | WNN3790-H005 | 0.538±0.035 | 75.62 | -22.45 |
| 123 | RUS0227-D005 | 0.54±0.022 | 78.05 | -5.69 |
| 124 | WNN2804-H009 | 0.54±0.105 | N.D. | 4.62 |
| 125 | WNN2412-H004 | 0.543±0.056 | 27.68 | 5.06 |
| 126 | WNN3989-H007 | 0.552±0.12 | 54.96 | -4.89 |
| 127 | WNN2548-H010 | 0.556±0.009 | N.D. | 26.72 |
| 128 | WNN0370-D008 | 0.566±0.03 | 63.92 | -12.77 |
| 129 | JK0771-B008 | 0.58±0.01 | 70.58 | -5.53 |
| 130 | WNN3317-B007 | 0.587±0.022 | 54.3 | -1.61 |
| 131 | WNN1444-C002 | 0.589±0.137 | 32.87 | -1.55 |
| 132 | WNN2231-D004 | 0.592±0.085 | N.D. | 4.09 |
| 133 | JK0468-F002 | 0.594±0.04 | 42.95 | -18.55 |
| 134 | JK1162-C006 | 0.597±0.074 | -21.27 | -15.44 |
| 135 | NCRW0109-A011 | 0.598±0.141 | 60.8 | -8.84 |
| 136 | WNN3886-G006 | 0.608±0 | 76.65 | -8.34 |
| 137 | WNN2531-B010 | 0.62±0.112 | N.D. | -0.49 |
| 138 | WNN2116-H010 | 0.623±0.037 | 47.38 | -0.29 |
| 139 | WNN2609-D002 | 0.639±0.024 | N.D. | 11.34 |
| 140 | WNN3979-D005 | 0.644±0.091 | 23.38 | -7.34 |
| 141 | WNN2604-H009 | 0.653±0.048 | 55.1 | 1.47 |
| 142 | WNN3476-D004 | 0.673±0.049 | 99.94 | -13.26 |
| 143 | JK0953-C005 | 0.697±0.074 | 5.57 | -13.49 |
| 144 | WNN3471-C011 | 0.701±0.022 | -96.8 | -3.07 |
| 145 | RUS0743-H008 | 0.705±0.047 | 93.91 | -10.03 |
| 146 | WNN3802-E008 | 0.711±0.055 | 74.57 | 12.99 |
| 147 | WNN3721-B007 | 0.728±0.042 | 67.2 | -15.66 |
| 148 | JK1173-G002 | 0.734±0.182 | -20.53 | -5.49 |
| 149 | WNN3265-B009 | 0.74±0.084 | 22.16 | 5.97 |
| 150 | WNN3956-C004 | 0.742±0.023 | -17.21 | -13.5 |
| 151 | WNN0320-B010 | 0.749±0.026 | -23.33 | 5.84 |
| 152 | WNN0204-D007 | 0.753±0.015 | 56.12 | -12.31 |
| 153 | WNN2753-F006 | 0.788±0.085 | 58.97 | -11.24 |
| 154 | WNN1299-C005 | 0.788±0.272 | 54.23 | 1.47 |
| 155 | JK3333-H005 | 0.802±0.106 | 39.19 | -15.09 |
| 156 | WNN0385-F010 | 0.81±0.109 | 43.24 | 9.63 |
| 157 | CB1582-F006 | 0.823±0.016 | 72.63 | 2.93 |
| 158 | CD0191-F004 | 0.827±0 | 99.44 | 15.79 |
| 159 | JK2340-F004 | 0.835±0.25 | 6.25 | -15.66 |
| 160 | CD2120-G005 | 0.845±0.087 | 52.35 | -16.07 |
| 161 | RD0034-A004 | 0.918±0.438 | -13.21 | -12.57 |
| 162 | WNN2290-B006 | 0.929±0.033 | N.D. | 4.67 |
| 163 | WNN0385-C008 | 0.948±0.067 | 62.36 | -0.51 |
| 164 | WNN0889-A010 | 0.967±0.037 | 57.12 | 1.37 |
| 165 | RUS0860-H004 | 16.42±0.281 | 99.85 | 99.22 |
| 166 | WNN5266-A005 | 3.881±0.266 | 62.8 | -15.64 |

N.D., not determined

**Table S2.** Summary of IC_50_, antiviral potencies and cytotoxicities of SARS-CoV-2 PL^pro^ inhibitors.

| No | Compound | IC50(μM) | Antiviral activity (%) | Cytotoxicity activity (%) |
| --- | --- | --- | --- | --- |
| 1 | WNN3369-C011 | 0.18±0.03 | 100 | 97.71 |
| 2 | NCRW0370-F002 | 0.28±0 | 100 | 95.14 |
| 3 | RUS0093-G006 | 0.32±0.06 | 100 | 96.6 |
| 4 | WNN3768-D008 | 0.5±0.02 | 100 | 99.41 |
| 5 | WNN1306-H005 | 0.56±0.08 | 18.81 | 14.19 |
| 6 | RUS0130-G006 | 0.78±0.19 | 100 | 97.44 |
| 7 | RUS1257-C009 | 0.81±0.07 | 100 | 92.76 |
| 8 | RUS0095-B004 | 0.84±0 | 99.23 | 55.53 |
| 9 | WNN1288-A004 | 0.89±0 | 53.14 | 11.76 |
| 10 | RUS1284-H004 | 0.9±0.03 | 100 | 97.21 |
| 11 | CDE0971-E008 | 0.91±0.07 | -44.64 | 20.61 |
| 12 | WNN3608-F003 | 1.04±0.12 | 100 | 97.46 |
| 13 | WNN1518-A007 | 1.3±0.39 | 70.78 | 47.69 |
| 14 | C0002-D002 | 1.63±0.60 | 31.66 | 2.26 |
| 15 | WNN0647-H011 | 1.7±0.18 | 100 | 98.74 |
| 16 | WNN1287-H011 | 1.71±0.05 | 100 | 86.95 |
| 17 | WNN1356-D011 | 1.71±0.07 | 11.83 | 11.34 |
| 18 | JK0949-H009 | 1.92±0.58 | 31.33 | -0.03 |
| 19 | CD3531-C011 | 1.98±0.23 | 75.07 | -14.28 |
| 20 | WNN3936-H009 | 2.06±0.08 | 100 | 89.93 |
| 21 | WNN2460-B003 | 2.08±0.3 | 24.34 | 20.23 |
| 22 | WNN1229-E002 | 2.22±0.37 | 97.68 | 44.32 |
| 23 | WNN0819-F004 | 2.31±0.38 | 87.83 | 36.3 |
| 24 | WNN3739-B007 | 2.47±0.33 | 100 | 97.79 |
| 25 | WNN3198-E003 | 2.72±0.39 | -5.35 | 6.51 |
| 26 | JK3137-F009 | 3.19±0.41 | 100 | 98.77 |
| 27 | WNN3367-E005 | 3.33±0.95 | 34.23 | -2.62 |
| 28 | RD0672-D010 | 3.37±0.39 | 74.91 | -9.17 |
| 29 | WNN0468-F009 | 3.75±0.95 | 96.47 | 51.28 |
| 30 | WNN3868-C011 | 4.32±0.41 | 100 | 95.78 |
| 31 | A0001-B006 | 4.42±0.21 | 100 | 99.3 |
| 32 | WNN3539-D004 | 4.64±0.62 | -8.87 | -2.51 |
| 33 | RD0042-H010 | 4.65±0.6 | 99.27 | 49.54 |
| 34 | WNN3632-A005 | 4.95±2.03 | -1.98 | -6.2 |
| 35 | WNN3823-C002 | 5.08±0.34 | 98.14 | 44.43 |
| 36 | WNN0649-F005 | 5.24±1.36 | 92.73 | 48.93 |
| 37 | WNN3805-A005 | 5.77±0.11 | 28.99 | -9.92 |
| 38 | WNN3374-B009 | 5.77±2.9 | 17.04 | 3.41 |
| 39 | JK0109-B011 | 6.01±0.02 | -7.89 | -7.31 |
| 40 | RD0063-D003 | 6.85±0.9 | 99 | 47.4 |
| 41 | WNN3936-D005 | 7.31±0.54 | 91.56 | 40.82 |
| 42 | RD0042-B011 | 7.39±1.8 | 70.8 | 0.39 |
| 43 | RD0063-E003 | 7.55±1.51 | 92.01 | 47.08 |
| 44 | RUS1119-D010 | 7.81±0.35 | 89.94 | 35.64 |
| 45 | WNN3477-D005 | 7.84±0.04 | -31.1 | -1.15 |
| 46 | WNN3273-H005 | 8.4±0.52 | 51.83 | -1.99 |
| 47 | LC0260-E007 | 8.46±1 | 51.18 | -6.98 |
| 48 | WNN3511-F004 | 8.51±0.7 | -2.61 | 0.82 |
| 49 | CD3226-D010 | 8.61±3.07 | 86.56 | -22.39 |
| 50 | WNN0482-D002 | 8.75±1.14 | 54.38 | 7.89 |
| 51 | WNN4116-F010 | 8.81±0.81 | -8.97 | -2.48 |
| 52 | WNN3285-B009 | 9.02±1.82 | -21.89 | 1.73 |
| 53 | WNN0331-G004 | 9.25±1.43 | -15.47 | 1.88 |
| 54 | RUS0858-H007 | 9.49±1.75 | -6.81 | 1.31 |
| 55 | C0012-F008 | 9.50±0.30 | 28.51 | -11.65 |
| 56 | CDE1323-H007 | 9.53±0.59 | 99.25 | 41.25 |
| 57 | PC0003-G004 | 9.82±1.10 | 100 | -17.2 |
| 58 | A0018-G010 | 9.92±0.05 | 42.72 | -14.12 |
| 59 | JK0109-H010 | 10.06±1.09 | 36.76 | -6 |
| 60 | WNN3516-D006 | 10.07±0.97 | -10.83 | -3.47 |
| 61 | WNN3910-E011 | 10.25±1.01 | 100 | 71.12 |
| 62 | CD1001-A006 | 10.33±3.22 | 39.64 | -12.07 |
| 63 | C0004-A006 | 10.49±2.22 | 74.19 | 13.32 |
| 64 | RD0042-C011 | 10.75±0.98 | 99.35 | 40 |
| 65 | NCRW0126-E006 | 10.82±0.53 | 99.99 | -14.45 |
| 66 | WNN0492-F006 | 11.14±0.82 | -10.32 | 2.94 |
| 67 | A0026-F005 | 11.18±0.18 | 83.36 | 8.77 |
| 68 | WNN0112-C003 | 11.33±0.19 | 100 | 99.06 |
| 69 | RD0042-A007 | 11.36±2.29 | 54.43 | 12.85 |
| 70 | CDE1324-E006 | 11.74±1.95 | 99.7 | 89.83 |
| 71 | C0015-D007 | 12.00±0.57 | 100 | 69.19 |
| 72 | PC0003-H006 | 12.34±2.97 | 61.55 | -25.08 |
| 73 | WNN3767-C011 | 12.52±0.87 | 86.37 | 5.45 |
| 74 | C0003-G011 | 12.75±0.93 | 100 | 43.7 |
| 75 | CDE0715-H003 | 12.84±3.86 | 49.64 | -20.44 |
| 76 | WNN0726-A006 | 12.94±0.25 | -17.33 | 7.38 |
| 77 | WNN3960-F006 | 13.1±0.22 | -10.53 | -3.52 |
| 78 | A0009-B011 | 13.28±0.69 | 100 | 32.75 |
| 79 | CD3562-H007 | 13.33±1.22 | 99.97 | 47.82 |
| 80 | CDE0715-B004 | 13.58±0.26 | 32.05 | -20.02 |
| 81 | PC0005-E011 | 15.29±7.34 | 93.7 | 38.45 |
| 82 | JK0946-H005 | 15.81±1.04 | 44.03 | -5.56 |
| 83 | C0007-B002 | 15.92±0.49 | 100 | 49.17 |
| 84 | C0003-F010 | 16.51±0.47 | 35.37 | -2.72 |
| 85 | WNN3286-E003 | 17±3.03 | -19.7 | -11.33 |
| 86 | JK0946-F007 | 17.01±0.15 | 39.88 | -4.63 |
| 87 | CDE0715-A006 | 17.25±1.12 | 29.85 | -20 |
| 88 | RUS0858-G007 | 17.57±1.53 | 71.47 | 13.62 |
| 89 | CDE0714-F008 | 17.8±2.63 | 36.77 | -16.03 |
| 90 | WNN3959-D005 | 17.89±5.71 | 80.94 | 27.6 |
| 91 | JK3137-G009 | 18±1.61 | 100 | 96.89 |
| 92 | JK3137-E009 | 18.03±0.69 | 99.96 | 71.07 |
| 93 | WNN2184-D009 | 18.05±2.55 | 22.97 | 0.34 |
| 94 | PC0001-C011 | 18.75±2.30 | 45.12 | -6.83 |
| 95 | WNN0391-G003 | 18.87±2.06 | 0.4 | 0.84 |
| 96 | WNN1259-B006 | 19±8.44 | 63.08 | 30.75 |
| 97 | RUS0746-G008 | 19.19±4.55 | 28.04 | -2.04 |
| 98 | WNN2217-F011 | 19.4±0.4 | 56.96 | 32.75 |
| 99 | A0021-F007 | 19.58±1.02 | 69.82 | -4.13 |
| 100 | WNN1560-B010 | 19.63±2.46 | -2.26 | 5.15 |
| 101 | CDE0715-G003 | 19.94±2.04 | 43.12 | -19.61 |
| 102 | PC0003-H010 | 20.43±0.92 | 50.98 | 27.51 |
| 103 | JK3137-H009 | 20.45±1.02 | 95.84 | 40.49 |
| 104 | JK0109-C011 | 21.19±1.79 | 30.8 | -9.42 |
| 105 | NCRW0216-B009 | 22.65±0.1 | 100 | 24.35 |
| 106 | CDE0715-E003 | 23.09±0.55 | 42.08 | -20.15 |
| 107 | C0007-C004 | 23.26±2.49 | 100 | 36.62 |
| 108 | RD1020-D003 | 23.57±0.28 | 82.97 | -5.6 |
| 109 | RUS0835-C002 | 24.7±4.21 | 100 | 44.75 |
| 110 | WNN3934-C011 | 24.9±3.86 | -6.75 | -8.97 |
| 111 | WNN1410-F008 | 25.73±4.37 | 99.78 | 93.25 |
| 112 | RUS0360-E010 | 27.73±5.8 | 50.83 | 15.19 |
| 113 | RUS0198-F003 | 28.2±0.98 | 50.98 | 0.21 |
| 114 | LC0015-A006 | 28.64±6.51 | 73.92 | 22.14 |
| 115 | C0012-E009 | 29.31±2.76 | 54.09 | -8.29 |
| 116 | WNN3450-D011 | 29.37±1.35 | 27.69 | 3.37 |
| 117 | RUS0001-G008 | 29.92±1.09 | 83.18 | -6.79 |
| 118 | WNN0640-F002 | 30.18±4.12 | -13 | 2.67 |
| 119 | WNN4032-D008 | 31.13±2.52 | -0.47 | 1.56 |
| 120 | JK0109-H005 | 31.14±1.26 | 10.75 | -4.08 |
| 121 | JK1163-A004 | 41.86±3.56 | 94.61 | 47.74 |
| 122 | JK0109-B004 | 43.02±0.92 | 40.8 | -7.5 |
| 123 | JK0108-D008 | 58.78±15.3 | 20.6 | -8.62 |

**Table S3.** Summary of IC_50_, antiviral potencies and cytotoxicities of representative inhibitors.

| No | Compound | IC_50_ for M^pro^ (μM) | IC_50_ for PL^pro^(μM) | Antiviral activity(%) | Cytotoxicity activity(%) |
| --- | --- | --- | --- | --- | --- |
| 1 | Compound **3a** | 0.5 ± 0.0 | 58.8 ± 15.3 | 20.60 | -8.62 |
| 2 | Compound **3b** | 21.8 ± 0.3 | 6.0 ± 0.0 | -7.89 | -7.31 |
| 3 | Compound **3c** | 8.7 ± 0.9 | 17.0 ± 0.2 | 39.88 | -4.63 |
| 4 | Compound **3d** | 4.2 ± 0.7 | 31.1 ± 1.3 | 10.75 | -4.08 |
| 5 | Compound **3e** | 42.8 ± 1.7 | 21.2 ± 1.8 | 30.80 | -9.42 |
| 6 | Compound **3f** | 9.7 ± 0.1 | 43.0 ± 0.9 | 40.80 | -7.50 |

**Table S4.** Data collection and refinement statistics. The highest resolution shell is shown in parentheses.

| ***Data collection*** | | |
| --- | --- | --- |
| **Space group** | *C2* | |
| **Cell dimensionsa, b, c (Å)** | 97.3, 82.9, 51.6 | |
| **α, β, γ (º)** | 90.0, 115.4, 90.0 | |
| **Resolution (Å)** | 50.0-1.80 (1.86-1.80) | |
| ***R_merge_*** | 2.2 (29.6) | |
| **Mean *I/σ(I)*** | 18.5 (2.3) | |
| **Completeness (%)** | 99.6(99.8) | |
| **Redundancy** | 2.0 (2.0) | |
| ***Refinement*** | | |
| **Resolution (Å)** | 50.0-1.80 | |
| **Number of reflections(test set)** | 34,224 (3432) | |
| ***R_work_ / R_free_*(%)** | 22.0 / 24.6 | |
| **Number of atoms**  **Protein**  **Ligand**  **Waters** | 2,370  46  263 |  |
| **Overall *B* values (Å^2^)**  **Protein**  **Ligand**  **Water** | 40.7  55.9  49.2 |  |
| **RMSD**  **Bond lengths (Å)**  **Bond angles (°)** | 0.016  1.39 | |
| **Ramachandran plot statistics (%)***  **Favored regions**  **Allowed regions**  **Disallowed regions** | 98.36  1.64  0.0 | |

*As defined in ProCheck.

**Table S5.** Summary of IC_50_ of SARS-CoV-2 M^pro^ inhibitors under different substrate concentrations.

| No | Compound | 1/2^a^ | 1 | 2 |
| --- | --- | --- | --- | --- |
|  |  |  | IC_50_ (μM) |  |
| 1 | WNN2048-F004 （compound 4a） | 0.32±0.01 | 0.35±0.11 | 0.33±0.07 |
| 4 | WNN3953-B007 （compound 5a） | 0.28±0.08 | 0.77±0.14 | 0.53±0.10 |
| 5 | WNN3989-H007 （compound 5b） | 0.11±0.01 | 0.18±0.01 | 0.18±0.07 |
| 6 | RUS0245-E010 （compound 5c） | 0.34±0.04 | 0.25±0.04 | 0.34±0.09 |
| 7 | WNN3797-H008 （compound 5d） | 0.67±0.28 | 0.56±0.19 | 0.55±0.02 |
| 8 | CDE0972-G011（compound 5e） | 2.17±0.20 | 2.86±0.53 | 3.50±0.42 |
| 9 | JK0108-D008 （compound 3a） | 1.70±0.93 | 4.37±0.84 | 5.83±1.68 |
| 10 | JK0109-B011（compound 3b） | 1.33±0.26 | 1.53±0.58 | 3.37±0.84 |
| 11 | JK0946-F007（compound 3c） | 3.56±0.62 | 9.67±3.64 | 8.97±2.32 |
| 12 | JK0109-H005（compound 3d） | 1.33±0.28 | 4.17±1.29 | 3.36±0.47 |
| 13 | JK0109-C011（compound 3e） | 2.30±0.96 | 16.10±3.07 | 17.76±3.25 |
| 14 | JK0109-B004（compound 3f） | 1.01±0.25 | 1.62±0.24 | 2.17±0.27 |

^a^ S/K_M_, theratioof substrate concentration and enzyme kinetic constant K_M_

**Table S6.** Summary of IC_50_ of SARS-CoV-2 PL^pro^ inhibitors under different substrate concentrations.

| No | Compound | 1/4 ^a^ | | 1/2 | | 1 | | 2 | 4 |
| --- | --- | --- | --- | --- | --- | --- | --- | --- | --- |
|  |  | IC_50_ (μM) | | | | | | | |
| 1 | JK0108-D008 （compound 3a） | 100.4±29.6 | 94.6±11.3 | | 102.5±1.2 | | 199.7±47.6 | | 132.9±14.7 |
| 2 | JK0109-B011（compound 3b） | 7.9±1.7 | 7.4±0.6 | | 7.3±0.1 | | 12.4±1.7 | | 10.15±1.0 |
| 3 | JK0946-F007（compound 3c） | 24.7±3.2 | 30.3±2.3 | | 43.9±9.0 | | 104.9±4.7 | | 201.8±50.9 |
| 4 | JK0109-H005（compound 3d） | 16.4±4.1 | 21.9±2.0 | | 33.0±17.9 | | 78.5±5.7 | | 91.6±8.9 |
| 5 | JK0109-C011（compound 3e） | 5.9±0.2 | 7.1±0.7 | | 9.4±2.9 | | 10.7±0.7 | | 18.6±2.7 |
| 6 | JK0109-B004（compound 3f） | 2.4±0.1 | 2.6±0.5 | | 5.9±0.6 | | 8.5±0.9 | | 18.3±1.0 |
| 7 | CD3531-C011 （compound 7a） | 1.5±0.4 | 1.5±0.0 | | 2.2±0.3 | | 2.4±0.2 | | 2.7±0.1 |
| 8 | CD3226-D010 （compound 7b） | 7.6±1.0 | 8.4±2.0 | | 10.8±2.1 | | 11.1±0.4 | | 12.5±0.3 |

^a^ S/K_M_, theratioof substrate concentration and enzyme kinetic constant K_M_
